# Periodontal Diseases and Adverse Pregnancy Outcomes: Review of Two Decades of Clinical Research

**DOI:** 10.3290/j.ohpd.b898969

**Published:** 2021-01-26

**Authors:** Zocko Ange Désiré Pockpa, Assem Soueidan, Nadin Thérèse Koffi-Coulibaly, Alexandre Limam, Zahi Badran, Xavier Struillou

**Affiliations:** a Assistant, Department of Periodontology, Faculty of Dental Surgery, University of Felix Houphouët Boigny, Ivory Coast. Idea, methodological design, definition of search strategy, search and selection of articles, data extraction, wrote and approved the manuscript.; b Professor, Department of Periodontology, Faculty of Dental Surgery, University of Nantes, France. Project supervisor, methodological design, definition of search strategy, synthesis of results, read and approved the manuscript.; c Senior Lecturer, Department of Periodontology, Faculty of Dental Surgery, University of Felix Houphouët Boigny, Ivory Coast. Read and approved the manuscript.; d Postgraduate Student, Department of Periodontology, Faculty of Dental Surgery, University of Nantes, France. Data extraction, quality assessement of included studies, read and approved the manuscript.; e Professor, Department of Periodontology, (CHU/Rmes Inserm U1229/UIC11), Faculty of Dental Surgery, University of Nantes, France; College of Dental Medicine, University of Sharjah, Sharja, UAE; Faculty of Dentistry, McGill University, Montreal, Canada. Quality assessement of included studies, read and approved the manuscript.; f Senior Lecturer, Department of Periodontology, Faculty of Dental Surgery, University of Nantes, France. Project supervisor, methodological design, definition of search strategy, synthesis of results, read and approved the manuscript.

**Keywords:** periodontal diseases, pregnancy, adverse pregnancy outcomes, mapping.

## Abstract

**Purpose:**

The aim of this study was to review the literature and chart the clinical studies that have focused on periodontal diseases and adverse pregnancy outcomes since 1996.

**Materials and Methods:**

Medline, Cinahl, and Cochrane databases were searched for original studies focused on pregnancy outcomes and periodontal status in humans. The most recent search was conducted on April 30, 2020.

**Results:**

Of the 633 articles identified, 232 articles (n = 119,774 participants) were selected for analysis. The majority of studies highlighted a statistically significant association between periodontal diseases and preterm birth (71 of 111 articles; 63.96%), low birth weight (46 of 64 articles; 71.87%), preterm low birth weight (29 of 49 articles; 59.18%), preeclampsia (31 of 45 articles; 68.89%) and other pregnancy complications, such as preterm, prelabor rupture of membranes (17 of 26 articles; 65.38%). Geographical analysis revealed that clinical studies were conducted in 51 countries, primarily in the United States (42 studies, 18.10%), Brazil (33 studies, 14.22%) and India (25 studies, 10.78%). Irrespective of geographical location, analysis showed various degrees of evidence of a relationship between periodontal diseases and adverse pregnancy outcomes.

**Conclusion:**

The majority of the studies found a statistically significant link between periodontal diseases and some complications of pregnancy. The strength of such a link varies according to type of study, type of variable and outcome measure selected.

Periodontal disease, such as gingivitis and periodontitis, is an immuno-inflammatory infection-induced condition affecting the tissues supporting the teeth (gingiva, periodontal ligaments, cementum, and alveolar bone).^28^ In susceptible patients, it results from a deficient host response following dysbiosis of the bacterial biofilm.^13^ If untreated, permanent loss of teeth may result, entailing adverse repercussions in terms of the individual’s general state of health.^19,46^ Indeed, various studies have demonstrated a link between periodontitis and conditions such as diabetes,^31^ rheumatoid arthritis,^5^ cardiovascular disorders,^39^ age-related macular degeneration,^44^ and erectile dysfunction.^48^

In 1996, Offenbacher^25^ first documented that pregnant women with periodontitis were at a 7.5-times higher risk of delivering preterm low birth weight infants than women with healthy periodontal tissue. In the wake of this initial clinical study, a large body of research work has addressed the relationship between periodontal diseases and obstetrical outcomes.^1,3,6,9,10,17,22-24,32,34,37,41^

The aim of this study was to review the literature and chart the clinical studies that focused on periodontal diseases and adverse pregnancy outcomes (APOs) over the two decades since Offenbacher’s publication.^25^

## Materials and Methods

### Search Strategy

**Fig 1:**
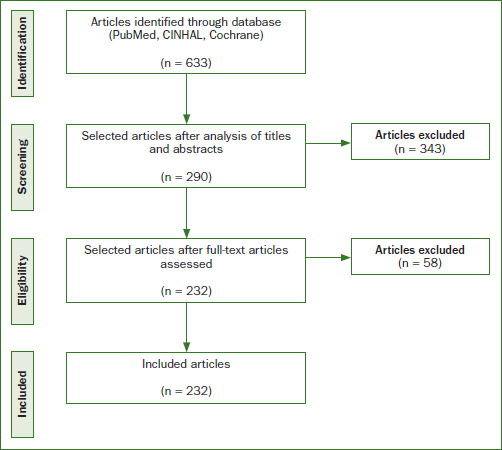
Flowchart of study selection.

The following databases were searched electronically: PubMed, CINAHL, and Cochrane (Fig 1). Published epidemiological and interventional studies suggesting a relationship between periodontal diseases and APOs were selected. The search was conducted using a combination of several key words. All of the references obtained were entered into Zotero 5.0 software (Center for History and New Media, George Mason University; Fairfax, VA, USA). The search targeted articles published in English or French, with the most recent search conducted on April 30, 2020.

### Inclusion Criteria

To be included, articles were required to have the following characteristics:

- based on original clinical studies (randomized controlled trial, not-randomized controlled trial, cohort study, case-controlled study, cross-sectional study);
- a focus on periodontal diseases and preterm birth (PTB) and/or low birth weight (LBW) and/or preterm low birth weight (PLBW) and/or preeclampsia (PREEC) and/or other pregnancy complications (OPC);
- published in English or French.

### Exclusion Criteria

Articles were excluded if they were:

- animal or in vitro studies;
- beyond the scope of the research;
- published in a language other than English or French;
- opinion-based articles, literature reviews, systematic reviews, or meta-analyses.

### Data Extraction

The following data were extracted from each article retrieved: name of principal author, year of publication, country of research, type of study, number of participants, type of obstetrical complication under investigation, assessment and diagnostic criteria for periodontal diseases, and main study findings and conclusions. The articles were then sorted into various groups according to the given obstetrical complication. If an article focused on several obstetrical complications, it was included in several groups.

## Results

**Fig 2:**
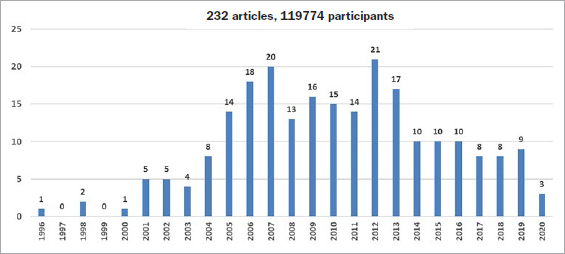
Distribution of trials by year.

A total of 633 potentially relevant articles were identified. After eliminating those which failed to meet the inclusion criteria outlined, 232 articles (n=119,774 participants) were included for analysis (Fig 2). The sample included 193 epidemiological studies and 39 interventional studies. About 10 articles were published each year on this topic in various journals (periodontology, gynecology, odontology, biology, etc).

**Fig 3:**
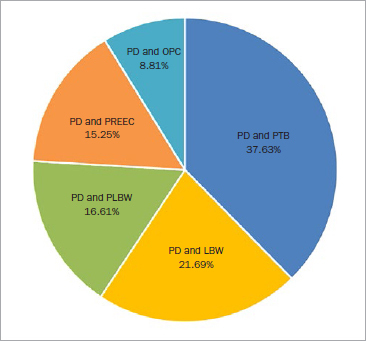
Obstetric complications investigated.

The obstetrical complications investigated were: PTB (111 articles, 37.63%), LBW (64 articles, 21.69%), PLBW (49 articles, 16.61%), PREEC (45 articles, 15.25%), and OPC (26 articles, 8.81%), such as preterm prelabor rupture of membranes, spontaneous abortion, fetal growth restriction, and stillbirth (Fig 3). The majority of studies highlighted a significant relationship between periodontal diseases and PTB (63.96%), LBW (71.87%), PLBW (59.18%), PREEC (68.89%), and OPC (65.38%) (Table 1).

**Table 1:** Results obtained by type of study and obstetrical outcomes

	RCT			Cohort			Case-control			Cross-sectional			Total		
	+	–	+/–	+	–	+/–	+	–	+/–	+	–	+/–	+	–	+/–
PD and PTB	13	9	0	24	12	0	28	14	1	6	4	0	71	39	1
PD and LBW	5	5	0	17	5	0	18	6	1	6	1	0	46	17	1
PD and PLBW	5	6	0	8	4	0	12	8	0	4	1	1	29	19	1
PD and PREEC	0	3	0	9	3	0	21	6	1	1	0	1	29	11	2
PD and OPC	1	1	0	7	6	0	5	2	0	1	0	0	7	7	0
	24	24	0	65	30	0	84	36	3	18	6	2	166	83	5

PD: periodontal diseases; PTB: preterm birth; LBW: low birth weight; PLBW: preterm low birth weight; PREEC: preeclampsia. OPC: other pregnancy outcomes; +: link; -: no link; +/-: reserved opinion; RCT: randomized contolled trial.

**Fig 4:**
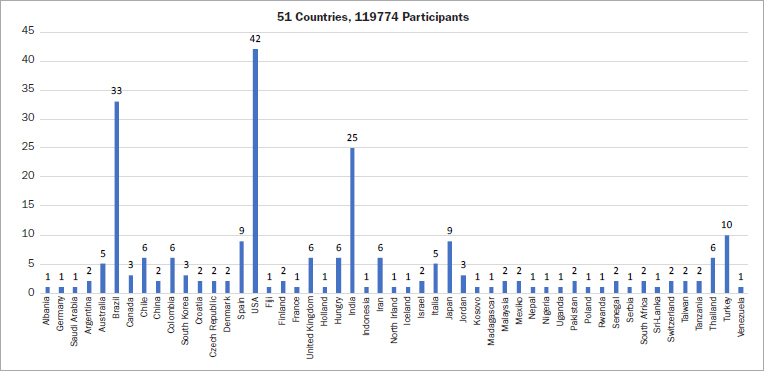
Distribution of trials by country.

**Fig 5:**
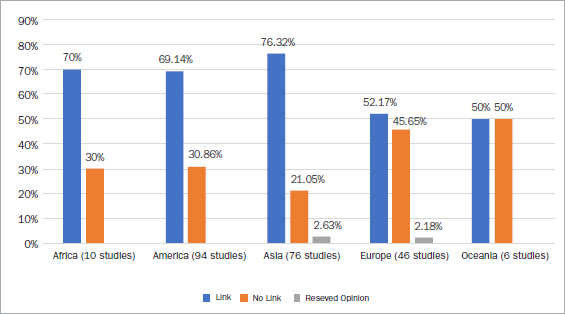
Distribution of trials results by continent.

A geographic analysis of the article search for this topic revealed that clinical studies were performed in 51 countries, mainly in the United States (42 studies, 18.10%), Brazil (33 studies, 14.22%), and India (25 studies, 10.78%) (Fig 4). The majority of studies were conducted in Africa (70%), North and South America (69.14%), Asia (76.32%) and demonstrate a statistically significant link between periodontitis and APOs (Fig 5). However, those conducted in Europe (52.17%) and Oceania (50%) show mixed results.

## Discussion

The purpose of the present work was to provide a review of the literature and chart clinical studies investigating a relationship between periodontal diseases and APO from 1996 onward. To the best of our knowledge, this review of the literature is the first of its kind to provide a worldwide overview of the clinical research work conducted on this topic during the last two decades. It also outlines how this research has evolved over time and documents findings from each continent.

Since 1996, 232 articles (about 10 articles/year) focusing on the relationship between periodontal diseases and APO have been published in PubMed, CINAHL, and Cochrane. At the outset, the studies focused solely on PTB and LBW. Then, over time, other obstetrical complications such as PREEC and OPC came to the fore of research work^10,34^ (Fig 3).

The majority of non-interventional studies (130/193 articles, 67.36%) have demonstrated a statistically significant link between periodontal diseases and APO (Table 1). This is consistent with the findings from several systematic reviews and/or meta-analyses previously conducted on this topic, demonstrating that 60% to 80% of the studies found a link between periodontal diseases and APO.^8,12,14,15,42,43,45,47^ In 2007, a systematic review by Xiong et al^47^ revealed that, in 29 of 44 (65.91%) studies published up to December 2006 (26 case-controlled studies, 13 cohort studies, and 5 controlled trials), a link was established between periodontal disease and an increased risk of APO. In 2016, Corbella et al^8^ issued the findings of a meta-analysis based on 22 case-controlled studies and cohorts published between 1965 and 2015. According to these findings, periodontal disease is a statistically significant risk factor for PTB (Odds Ratio [OR]=1.61, CI [confidence interval] 95% 1.33–1.95), for LBW (OR=1.65; CI 95% 1.27–2.14), and for PLBW (OR=3.44, CI 95% 1.34–8.80). Similar findings were documented in a 2013 systematic review by Ide and Papapanou.^15^ That same year, a systematic review by Guirassy et al^12^ highlighted that 10 of 13 studies (76.92%) found a link between periodontal diseases and the incidence of PREEC. Similar conclusions were drawn in meta-analyses by Wei et al^45^ and Huang et al.^14^ In short, the majority of the non-intervention studies conducted over the two decades confirms a link (however weak or strong) between periodontal diseases and APO.

However, the results of interventional studies have shown to a lesser degree that periodontal treatment administered to pregnant women effectively prevents APOs,^2,11,17, 18,33,36^ while others reach the opposite conclusion.^21,26,29^ In a total of 39 interventional studies, 24 (61.54%) identified beneficial effects of periodontal treatment administered during pregnancy on APOs (Table 1). Condylis et al^7^ reported the same observation: 9 of 15 (60%) RCTs found a significant reduction in complications of pregnancy after periodontal treatment. However, a meta-analysis in 2010 by Uppal et al^40^ and another by Polyzos et al^30^ suggested that periodontal treatment had no effect on pregnancy outcomes. They indicated numerous sources of bias that may have affected the findings of those studies which concluded that periodontal treatment had a beneficial effect on APO. For instance, they underlined numerous disparities in the inclusion criteria. Similarly, the meta-analysis (15 RCT) by Iheozor-Ejiofor et al^16^ found insufficient evidence to conclude that periodontal treatment administered during pregnancy was instrumental in reducing PTB rates (relative risk = 0.87; 95% CI: 0.70–1.10). Nonetheless, given the timescale of recruitment and administration of periodontal treatment in these clinical trials (for the most part during the second and third trimesters of pregnancy), the possibility that earlier treatment (during the first trimester or even prior to conception) could have had a positive impact on the prevention of some types of obstetrical complications cannot be ruled out.^16^

All the data under discussion were collected from various countries throughout the world, encompassing diverse sanitary, cultural, and socioeconomic conditions. In the Americas and Asia, where the majority of the trials on this topic were undertaken (94 and 76 studies respectively), approximately 3 of 4 studies found evidence of a statsitically significant link between periodontal diseases and APO. This rate is lower in Europe, where only 52.17% of the studies (1 in 2) made a significant connection between periodontal diseases and APO. In Oceania and Africa, little data are available in this regard. However, the majority of the studies substantiated a relationship between periodontal diseases and APO (Fig 5).

Differents definitions have been used to define the presence of periodontitis. This observation has been made by several authors of similar studies who believe that this methodological heterogeneity constitutes a real obstacle for the comparison of studies between them and could influence the results obtained.^12,15,16,38,43^ The introduction by consensus reports of the new classification of periodontitis should allow a harmonization of the diagnostic and evaluation criteria of the periodontal status for future investigations.^4,27^

Unlike periodontitis, the studies found identical criteria for defining premature deliveries, low birth weight newborns and preeclampsia.

The abundance of findings from these past 20 years of research have led to a positive impact on the various health policies of several countries, as prenatal consultation with an oral health specialist has become a standard part of healthcare for pregnant women.^20,35^ In Canada, for example, an initial appointment with a dental surgeon is recommended from the start of pregnancy for removal of plaque where required. A second appointment is then scheduled during the second trimester of pregnancy for implementation of the treatment agreed upon at the first appointment.^35^ In France, an oral-health prevention program for pregnant women, implemented by the French national healthcare insurance provider (CPAM), has been operating since 2013.^20^ It comprises a free preventive oral-health checkup with a dental surgeon for all pregnant women in the fourth month of pregnancy. Its main purpose is to screen for periodontal diseases during pregnancy, with a particular focus on women at risk of PTB. This initiative is consistent with good practice guidelines and should be recommended for all pregnant women.

## Conclusion

Since 1996, periodontal disease has been considered a potential risk factor for the occurrence of APOs. Regardless of the continent, the majority of epidemiological studies undertaken have identified a significant yet tenuous link between periodontal diseases and certain complications of pregnancy. The strength of such a link varies according to type of study, type of variable, and outcome measure selected. Whether periodontal treatment has a beneficial effect on reducing the risk of adverse pregnancy outcomes, however, remains debatable.

## References

[ref1] Agueda A, Ramón JM, Manau C, Guerrero A, Echeverría JJ (2008). Periodontal disease as a risk factor for adverse pregnancy outcomes: a prospective cohort study. J Clin Periodontol.

[ref2] Albert DA, Begg MD, Andrews HF, Williams SZ, Ward A, Conicella ML, Rauh V, Thomson JL, Papapanou PN (2011). An examination of periodontal treatment, dental care, and pregnancy outcomes in an insured population in the United States. Am J Public Health.

[ref3] Boggess KA, Berggren EK, Koskenoja V, Urlaub D, Lorenz C (2013). Severe preeclampsia and maternal self-report of oral health, hygiene, and dental care. J Periodontol.

[ref4] Chapple ILC, Mealey BL, Van Dyke TE, Bartold PM, Dommisch H, Eickholz P, Geisinger ML, Genco RJ, Glogauer M, Goldstein M (2018). Periodontal health and gingival diseases and conditions on an intact and a reduced periodontium: Consensus report of workgroup 1 of the 2017 World Workshop on the Classification of Periodontal and Peri-Implant Diseases and Conditions. J Periodontol.

[ref5] Cheng Z, Meade J, Mankia K, Emery P, Devine DA (2017). Periodontal disease and periodontal bacteria as triggers for rheumatoid arthritis Best Practice & Research. Clin Rheumatol.

[ref6] Cisse D, Diouf M, Faye A, Diadhiou MF, Tal-Dia A (2015). Periodontal Disease of Pregnant Women and Low Weight Newborn in Senegal: A Case-Control Study. Open J Epidemiol.

[ref7] Condylis B, Le Borgne H, Demoersman J, Campard G, Philippe H-J, Soueidan A (2013). [Interest of periodontitis screening and treatment in pregnancy: systematic review]. Journal De Gynecologie, Obstetrique Et Biologie De La Reproduction.

[ref8] Corbella S, Taschieri S, Del Fabbro M, Francetti L, Weinstein R, Ferrazzi E (2016). Adverse pregnancy outcomes and periodontitis: A systematic review and meta-analysis exploring potential association. QI.

[ref9] Dasanayake AP, Gennaro S, Hendricks-Muñoz KD, Chhun N (2008). Maternal periodontal disease, pregnancy, and neonatal outcomes MCN. Am J Maternal Child Nurs.

[ref10] Farrell (née Moore) S, Ide M, Wilson RF (2006). The relationship between maternal periodontitis, adverse pregnancy outcome and miscarriage in never smokers. J Clin Periodontol.

[ref11] Gomes-Filho IS, Cruz SS, Costa M da CN, Passos JS, Cerqueira EMM, Sampaio FP, Pereira EC, Miranda LF (2010). Periodontal therapy and low birth weight: preliminary results from an alternative methodologic strategy. J Periodontol.

[ref12] Guirassy M (2016). Relation entre maladie parodontale et risque de survenue d’une pré-éclampsie : revue systematique. Rev Col Odonto-Stomatol Afr Chir Maxillo-Fac.

[ref13] Hajishengallis G (2015). Periodontitis: from microbial immune subversion to systemic inflammation. Nature Rev Immunol.

[ref14] Huang X, Wang J, Liu J, Hua L, Zhang D, Hu T, Ge Z-L (2014). Maternal periodontal disease and risk of preeclampsia: a meta-analysis. J Huazhong Univ Sci Technol Med Sci.

[ref15] Ide M, Papapanou PN (2013). Epidemiology of association between maternal periodontal disease and adverse pregnancy outcomes –systematic review. J Periodontol.

[ref16] Iheozor-Ejiofor Z, Middleton P, Esposito M, Glenny A-M (2017). Treating periodontal disease for preventing adverse birth outcomes in pregnant women. Cochrane Database Syst Rev.

[ref17] Jeffcoat M, Parry S, Gerlach RW, Doyle MJ (2011). Use of alcohol-free antimicrobial mouth rinse is associated with decreased incidence of preterm birth in a high-risk population. Am J Obstet Gynecol.

[ref18] Jeffcoat MK, Jeffcoat RL, Tanna N, Parry SH (2014). Association of a common genetic factor, PTGER3, with outcome of periodontal therapy and preterm birth. J Periodontol.

[ref19] Kinane D, Bouchard P, Group E of European Workshop on Periodontology (2008). Periodontal diseases and health: Consensus Report of the Sixth European Workshop on Periodontology. J Clin Periodontol.

[ref20] Legifrance (2013). Arrêté du 26 novembre 2013 portant approbation de l’avenant n° 3 à la convention nationale organisant les rapports entre les chirurgiens-dentistes et l’assurance maladie signé le 31 juillet.

[ref21] Macones GA, Parry S, Nelson DB, Strauss JF, Ludmir J, Cohen AW, Stamilio DM, Appleby D, Clothier B, Sammel MD (2010). Treatment of localized per not reduce the occurrence of preterm birth: results from the Periodontal Infections and Prematurity Study (PIPS). Am J Obstet Gynecol.

[ref22] Meqa K, Dragidella F, Disha M, Sllamniku-Dalipi Z (2017). The association between periodontal disease and preterm low birthweight in Kosovo. Acta Stomatolog Croat.

[ref23] Nabet C, Lelong N, Colombier M-L, Monsarrat P, Vergnes J-N, Sixou M, Musset A-M, Goffinet F, Kaminski M (2014). Parodontite maternelle et causes d’accouchement prématuré - Étude cas-témoins EPIPAP. Actualités Odonto-Stomatologiques.

[ref24] Nabet C, Lelong N, Colombier M-L, Sixou M, Musset A-M, Goffinet F, Kaminski M, for the Epipap Group (2010). Maternal periodontitis and the causes of preterm birth: the case–control Epipap study. J Clin Periodontol.

[ref25] Offenbacher S (1996). Periodontal diseases: pathogenesis. Ann Periodontol.

[ref26] Oliveira AMSD, Oliveira PAD de, Cota LOM, Magalhães CS, Moreira AN, Costa FO (2011). Periodontal therapy and risk for adverse pregnancy outcomes. Clin Oral Investig.

[ref27] Papapanou PN, Sanz M, Buduneli N, Dietrich T, Feres M, Fine DH, Flemmig TF, Garcia R, Giannobile WV, Graziani F (2018). Periodontitis: Consensus report of workgroup 2 of the 2017 World Workshop on the Classification of Periodontal and Peri-Implant Diseases and Conditions. J Periodontol.

[ref28] Pihlstrom BL, Michalowicz BS, Johnson NW (2005). Periodontal diseases. Lancet.

[ref29] Pirie M, Linden G, Irwin C (2012). Intrapregnancy non-surgical periodontal treatment and pregnancy outcome: a randomized controlled trial. J Periodontol.

[ref30] Polyzos NP, Polyzos IP, Zavos A, Valachis A, Mauri D, Papanikolaou EG, Tzioras S, Weber D, Messinis IE (2010). Obstetric outcomes after treatment of periodontal disease during pregnancy: systematic review and meta-analysis. BMJ (Clinical research).

[ref31] Preshaw PM, Alba AL, Herrera D, Jepsen S, Konstantinidis A, Makrilakis K, Taylor R (2012). Periodontitis and diabetes: a two-way relationship. Diabetolog.

[ref32] Rakoto-Alson S, Tenenbaum H, Davideau J-L (2009). Periodontal diseases, preterm births, and low birth weight: findings from a homogeneous cohort of women in Madagascar. J Periodontol.

[ref33] Reddy BVR, Tanneeru S, Chava VK (2014). The effect of phase-I periodontal therapy on pregnancy outcome in chronic periodontitis patients. J Obstet Gynaecol.

[ref34] Riché EL (2017). Periodontal disease increases the risk of preterm delivery among preeclamptic women [cited. https://www.ncbi.nlm.nih.gov/pubmed/16013222.

[ref35] Ross L (2016). La santé dentaire des femmes durant la grossesse - Api Laval apigroupe [Internet]. https//apigroupe.com/des-dents-en-sante-pendant-la-grossesse-5- choses-a-surveiller/.

[ref36] Sant’Ana ACP, Campos MR de, Passanezi SC, Rezende MLR de, Greghi SLA, Passanezi E (2011). Periodontal treatment during pregnancy decreases the rate of adverse pregnancy outcome: a controlled clinical trial. J Appl Oral Sci.

[ref37] Soroye M, Ayanbadejo P, Savage K, Oluwole A (2015). Association between periodontal disease and pregnancy outcomes. Odonto-Stomatologie Tropicale [Tropical Dent J].

[ref38] Teshome A, Yitayeh A (2016). Relationship between periodontal disease and preterm low birth weight: systematic review. Pan African Med J.

[ref39] Tonetti MS, Van Dyke TE (2013). Periodontitis and atherosclerotic cardiovascular disease: consensus report of the Joint EFP/AAP Workshop on Periodontitis and Systemic Diseases. J Periodontol.

[ref40] Uppal A, Uppal S, Pinto A, Dutta M, Shrivatsa S, Dandolu V, Mupparapu M (2010). The effectiveness of periodontal disease treatment during pregnancy in reducing the risk of experiencing preterm birth and low birth weight: a meta-analysis. J Am Dent Assoc.

[ref41] Usin MM, Menso J, Rodríguez VI, González A, Tabares S, Parodi R, Sembaj A (2016). Association between maternal periodontitis and preterm and/or low birth weight infants in normal pregnancies. J Maternal-Fetal Neonatal Med.

[ref42] Vergnes J-N, Sixou M (2007). Preterm low birth weight and maternal periodontal status: a meta-analysis. Am J Obstetrics and Gynecology.

[ref43] Vettore MV, Lamarca G de A, Leão ATT, Thomaz FB, Sheiham A, Leal M do C (2006). Periodontal infection and adverse pregnancy outcomes: a systematic review of epidemiological studies. Cadernos De Saude Publica.

[ref44] Wagley S, Marra KV, Salhi RA, Gautam S, Campo R, Veale P, Veale J, Arroyo JG (2015). Periodontal disease and age-related macular degeneration: Results From the National Health and Nutrition Examination Survey III. Retina.

[ref45] Wei B-J, Chen Y-J, Yu L, Wu B (2013). Periodontal disease and risk of preeclampsia: a meta-analysis of observational studies. PLoS One.

[ref46] Wolf DL, Lamster IB (2011). Contemporary concepts in the diagnosis of periodontal disease. Dent Clin North Am.

[ref47] Xiong X, Buekens P, Fraser WD, Beck J, Offenbacher S (2006). Periodontal disease and adverse pregnancy outcomes: a systematic review. BJOG.

[ref48] Zuo Z, Jiang J, Jiang R, Chen F, Liu J, Yang H, Cheng Y (2011). Effect of periodontitis on erectile function and its possible mechanism. J Sex Med.

